# Targeting AP-1 transcription factors by CRISPR in the prostate

**DOI:** 10.18632/oncotarget.27997

**Published:** 2021-09-14

**Authors:** Maria Riedel, Huiqiang Cai, Iben C. Stoltze, Mikkel H. Vendelbo, Erwin F. Wagner, Latifa Bakiri, Martin K. Thomsen

**Affiliations:** ^1^Department of Clinical Medicine, Aarhus University, Aarhus, Denmark; ^2^Department of Biomedicine, Aarhus University, Aarhus, Denmark; ^3^Department of Nuclear Medicine & PET Centre, Aarhus University Hospital, Aarhus, Denmark; ^4^Laboratory Genes and Disease, Department of Dermatology, Medical University of Vienna (MUV), Vienna, Austria; ^5^Laboratory Genes and Disease, Department of Laboratory Medicine, Medical University of Vienna (MUV), Vienna, Austria; ^6^Aarhus Institute of Advanced Studies (AIAS), Aarhus University, Aarhus, Denmark

**Keywords:** AP-1, prostate cancer, CRISPR, mouse models, AAV

## Abstract

Prostate cancer is the second most diagnosed cancer in men. It is a slow progressing cancer, but when the disease reaches an advanced stage, treatment options are limited. Sequencing analyses of cancer samples have identified genes that can potentially drive disease progression. We implemented the CRISPR/Cas9 technology to simultaneously manipulate multiple genes in the murine prostate and thus to functionally test putative cancer driver genes *in vivo*. The activating protein-1 (AP-1) transcription factor is associated with many different cancer types, with the proto-oncogenes JUN and FOS being the two most intensely studied subunits. We analyzed expression of FOS and JUNB in human prostate cancer datasets and observed decreased expression in advanced stages. By applying CRISPR/Cas9 technology, the role of these two transcription factors in prostate cancer progression was functionally tested. Our data revealed that loss of either *JunB* or *Fos* in the context of *Pten* loss drives prostate cancer progression to invasive disease. Furthermore, loss of *Fos* increases Jun expression, and CRISPR inactivation of *Jun* in this context decreases cell proliferation. Overall, these *in vivo* studies reveal that *JunB* and *Fos* exhibit a tumor suppressor function by repressing invasive disease, whereas Jun is oncogenic and increases cell proliferation. This demonstrates that AP-1 factors are implicated in prostate cancer progression at different stages and display a dual function as tumor suppressor and as an oncogene in cancer progression.

## INTRODUCTION

Prostate cancer (PCa) is the second-most diagnosed cancer in men worldwide and numbers are expected to rise [1]. PCa is a slow progressing disease and the majority of cases are indolent. In contrast, metastatic PCa is a lethal disease with limited treatment options and a short survival [2, 3]. The molecular insights to PCa have revealed alterations in the classical pathways, such as PI3K and TP53, but the heterogeneity of PCa complicates the discovery and validation of unique gene alterations [4, 5]. While genome sequencing of PCa samples has revealed many potential driver mutations, their implication in PCa progression remains to be assessed using *in vitro* and *in vivo* model systems. Fortunately, the discovery of the CRISPR/Cas system to engineer specific gene alterations has permitted a great improvement of disease modelling using human cancer cell lines and mice.

Genetically engineered mouse models (GEMM) are essential to study gene function in PCa [6–8]. CRISPR/Cas system has improved the gene editing toolbox in numerous *in vivo* models, with the murine system being the most prevalent [9, 10]. We applied the CRISPR/Cas9 technology to study gene alterations in the murine prostate using orthotopic viral delivery of multiplexed sgRNAs, targeting multiple genes to evaluate their functions in PCa progression [11–13]. This method ensures simultaneous, multiplexed gene editing in adult mice and therefore bypassing timely breeding schemes. Another advantage of this method is the targeting of only few cells in the prostate epithelium, which allows tumor cells to clonally expand, similar to human cancer initiation [11].

We implemented CRISPR/Cas9 technology *in vivo* to analyze the role of the AP-1 subunits in prostate cancer progression. The activating protein-1 (AP-1) transcription factor is implicated in multiple biological processes from development, cell homeostasis, inflammation and cancer biology [14, 15]. The dimeric complex comprises basic region-leucine zipper (bZIP) proteins of the JUN, FOS, ATF, and MAF gene families. In cancer, AP-1 subunits exhibit multiple functions as oncogenes or tumor suppressors, depending on the specific context [15]. JUN, FOS, JUNB and JUND are implicated in PCa, together with JUN N-terminal kinase (JNK) [8, 16–20]. We initially sought to assess the function of FOS in PCa, as this subunit has been reported to be up- and downregulated in PCa [16, 21, 22] with no clear functional evaluation. For this, we applied CRISPR-mediated gene editing to generate loss of function of Fos as well as JunB and Jun in PCa, in the context of one of the major PCa mutation Pten.

### Research perspective

Different AP-1 genes have been associated with PCa [21]. First, we analyzed *FOS* expression at different stages of PCa from publicly available datasets. Our analyses revealed that *FOS* expression is decreased in advanced disease and further downregulated in metastatic PCa [11]. This indicated that *FOS* acts as a tumor suppressor gene in PCa progression. To functionally study the implication of Fos in PCa, we implemented the CRISPR/Cas9 technique and we altered *Fos* in combination with *Pten* in adult murine prostate. Loss of PTEN in human PCa occurs frequently and results in hyperactive cell division by increased phosphorylated AKT. Similar activation of p-Akt is seen in the murine prostate when Pten is mutated [23]. In this setting, we altered *Fos* in the murine prostate in combination with *Pten*. Intriguingly, loss of *Fos* increased cell proliferation and lead to an invasive tumor, whereas inactivation of *Pten* led to lesions that remained indolent and classified as high grade PIN [11]. This showed that *Fos* is indeed a tumor suppressor gene in prostate cancer. Further work is needed to show, whether *Fos* amplification can also drive cancer progression and if Fos could act both as oncogene and tumor suppressor gene in PCa.

To gain insight into the molecular consequences of the loss of *Fos*, we inactivated FOS by CRISPR/Cas9 in human prostate cancer cell lines. FOS depletion in the benign human prostate cell line BPH1 led to impaired proliferation and RNAseq analysis revealed alterations in multiple pathways. Interestingly, loss of *FOS* deregulated other AP-1 subunits, such as *JUN*. *JUN* expression was increased, and this was confirmed in the metastatic cell line DU145. Similarly, increased Jun expression was also found in the murine prostate, when *Fos* was depleted, indicating a conserved balance between the expression of *FOS* and *JUN* in prostate epithelial cells. Increased *JUN* expression has been shown to drive cancer progression, including PCa [15, 21]. We speculate that loss of *Fos* leads to increased *Jun* expression and drives cancer progression to an invasive disease with increased proliferation. Therefore, *Jun* was depleted in the murine prostate by CRISPR/Cas9 in combination with loss of *Fos* and *Pten*. By introducing three sgRNAs in the AAV construct, we generated triple-deficient prostate lesions. While the loss of *Jun* in combination with *Fos* and *Pten* decreased cell proliferation, the lesions still progressed to an invasive cancer [11]. This shows that *Jun* regulates proliferation, but that *Fos* is a gate keeper for the development of invasive prostate cancer independently of Jun.

**Figure 1:**
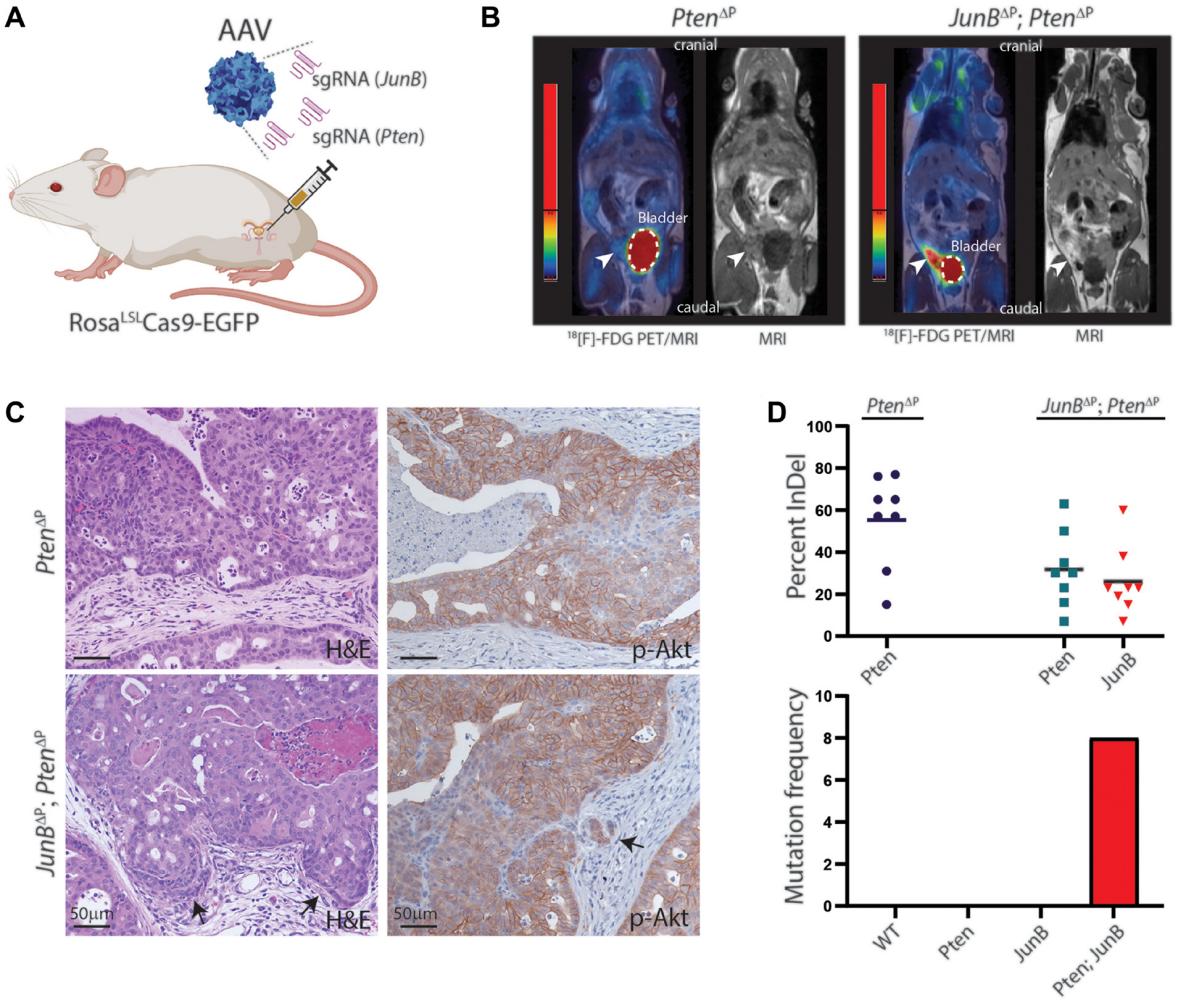
Loss of JunB and Pten in the murine prostate epithelium by CRISPR/Cas9. (**A**) Illustration of orthotopic AAV delivery of specific sgRNAs to the murine prostate of a Cas9 transgenic mouse. (**B**) Prostate cancer visualized by PET/MRI scanning using an 18[F]-FDG tracer to display enhanced glucose metabolism. Representative images of a Pten^∆P^ and a JunB^∆P^/Pten^∆P^ mouse 8 months post-injection. The white dashed lines marks the bladder and the arrowheads indicate enhanced FDG signal in the prostate (*n* ≥ 3). (**C**) H&E and IHC staining for p-Akt on FFPE tissue sections from the anterior prostate of Pten^∆P^ and JunB^∆P^/Pten^∆P^ mice 9 months post-injection. Arrows indicate invasive cells (*n* = 5). (**D**) Sanger sequencing data from tumor tissue biopsies analyzed using ICE (Synthego) 9 months post-injection. Gene editing efficiencies are plotted by out-of-frame InDel frequencies 9 months post-injection and for co-occurrences of multiple mutations (*n* = 8).

We previously showed that JUNB is a tumor suppressor gene by analyzing human samples and using GEMM, where JunB and Pten were inactivated by the Cre/lox technology in the whole prostate since week 4 [8]. To compare the CRISPR/Cas9 to the Cre/lox system for induction of PCa, we targeted *JunB* in combination with *Pten* by specific designed sgRNAs. By using AAV particles, the sgRNAs and a Cre recombinase were delivered directly to the anterior prostate lobe of 8-10 weeks old Cas9 transgenic mice by orthotopic injections [9] (Figure 1A). Cancer initiation and progression were followed by MRI scanning (Figure 1B) and the prostates were analyzed 3 and 9 months after initiation. Histological examination revealed transformed areas in the mouse prostate with high-grade prostatic intraepithelial neoplasia (PIN). Immunohistochemistry staining confirmed increased levels of p-Akt, indicating loss of Pten (Figure 1C). These data revealed that the CRISPR/Cas9 system in combination with AAV delivery of the sgRNA, is sufficient to alter gene expression in the murine prostate and led to comparable result as “classical” Cre/lox technology.

CRISPR-induced mutations can be analyzed by sequencing the sgRNA genomic target region. As CRISPR induces mutations by non-homologous end joining (NHEJ), different mutation profiles will occur with deletions and insertions (Indel) at the target site [9–11]. Analysis of the tumor samples reveals the presence of wild-type cells vs. cancer cells. Furthermore, the mutation profile indicates if the cancer is mono- or poly-clonal by assessing the different occurrences of Indels. Analysis of the mutations in *JunB* and *Pten* from the murine prostate confirmed loss of *JunB* and *Pten* in the majority of samples (Figure 1D). In addition, these data show that CRISPR-induced mutations create an unique fingerprint to the tumor, which is useful to follow and analyze the tumor evolution, which is not possible with the Cre/lox technology where all cells are mutated in the same way.

### Reflection

AP-1 transcription factor complexes intersect with numerous oncogenic signaling pathways and interfere with proliferation, invasion and metastasis formation [15, 24]. FOS is primarily associated with a pro-oncogenic activity and is commonly found overexpressed in tumors with poor prognosis [21, 25]. We demonstrated a tumor suppressor function of FOS in PCa by analyzing expression data from publicly available datasets. Decreased expression of other AP-1 subunits and pathways has been shown in advanced prostate cancer for JUNB and the c-Jun N-terminal kinase (JNK) [8, 19]. This shows that AP-1 signaling in prostate cancer is highly complex, as some subunits show opposite functions. Appropriate activation of AP-1 transcription factor complexes depends on a finely-tuned balance between every single member. Even though some of the dimeric subunits might share similar functions [15, 26], the loss of one factor affects expression of other subunits, as we have shown [11]. The altered expression of one subunit will not only influence the expression of other AP-1 members, but also their downstream target genes, resulting in a complex phenotype.

In our CRISPR/Cas9 mouse model, tumorigenesis is initiated by clonal expansion of single, edited cells that face natural selection pressure. Based on this model, we could show that the additional deletion of *Fos* in *Pten*-deficient epithelial cells led to increased proliferation and local invasion when compared to *Pten* KO tumors. Our findings revealed that *Jun* expression was increased in *Fos* deficient samples and by including sgRNA targeting *Jun,* we could subsequently address the function of this subunit in PCa. This shows an advantage of the CRISPR system, as we could bypass timely intercrossing of different mouse strains and rapidly perform functional assessment of *Jun* loss in the context of *Fos* and *Pten* deficient. AP-1 transcription factors are generally considered as oncogenes. We have shown that loss of *JunB* or *Fos* in combination with Pten-deficiency drives prostate cancer progression to an invasive disease. Hereby, we have delineated that JunB and Fos exhibit tumor suppressor function in PCa, while Jun is oncogenic in the context of Fos loss. Future work will reveal additional molecular mechanisms that are regulated by the AP-1 transcription complex and identify druggable targets, to overcome PCa progression to aggressive disease. Our studies further show that implication of AP1-factors in prostate cancer is complex and therefore targeting these factors with a broad inhibitor or agonist can have unforeseen consequences. It is therefore crucial to delineate the expression and function of each subunit before targeting the pathway. Further work is needed to thoroughly understand the function of AP-1 before therapeutically targeting AP-1 or pathways upstream and/or downstream of AP-1 in patients.

**Figure 2:**
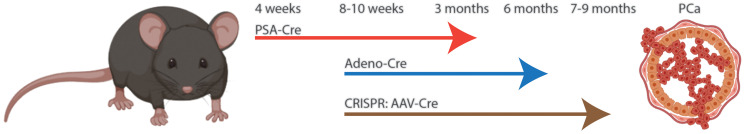
Comparison of Cre/lox to CRISPR induced prostate cancer in mouse. JunB and Pten were targeted simultaneously in the prostate epithelia by conditional deletions or CRISPR. Interbreeding of two strains, JunB^flox/flox^ and Pten^flox/flox^ with PSA-cre results in invasive prostate cancer at 3 months of age (C57bl/6x129). Delivery of Adeno virus for expression of Cre to the JunB^flox/flox^; Pten^flox/flox^ at 8 weeks of age results in invasive prostate cancer at 6 months (C57bl/6x129). Use of AAV to deliver sgRNA and Cre to the prostate of Rosa Lox-STOP-Lox Cas9-EGFP (C57bl/6) mice at 8-10 weeks of age led to invasive prostate cancer at 7–9 months of age.

We and others have bypassed the classical Cre-expressing transgenes by applying orthotopic delivery of adeno- or lentivirus expressing Cre to somatic cells [27, 8]. By targeting *JunB* in the mouse prostate by CRISPR, we could compare the timeline and results with gene inactivation using the Cre/lox system. We have previously shown that transgenic PsaCre expression in JunB; Pten floxed mice results in invasive tumors at 3 month of age, whereas Cre delivery to the prostate of adult JunB; Pten floxed mice by adeno virus injection results in PCa four months later [8]. After CRISPR induced mutations, we observed invasive PCa 6 months after orthotopic delivery (Figure 2). These results show, that cancer development is slower in adult mice, but is occurring with similar frequency even in the context of different genetic backgrounds. Overall, the CRISPR/Cas9 model established by us is advantageous over GEMM to study cancer, as it allows clonal expansion of transformed cells and because multiple mutations can be introduced simultaneously in somatic cells. Importantly, functional mechanistic studies can be performed without the need of time consuming intercrosses.
